# Rural-Urban Disparities in Diagnosis of Early-Onset Dementia

**DOI:** 10.1001/jamanetworkopen.2022.25805

**Published:** 2022-08-05

**Authors:** Wendy Y. Xu, Jeah Jung, Sheldon M. Retchin, Yiting Li, Soumyadipta Roy

**Affiliations:** 1 Division of Health Services Management and Policy, College of Public Health, the Ohio State University, Columbus; 2 Department of Health Administration and Policy, George Mason University, Fairfax, Virginia; 3 Division of General Internal Medicine, Department of Internal Medicine, College of Medicine, The Ohio State University, Columbus; 4 Department of Health Policy and Administration, College of Health and Human Development, Pennsylvania State University

## Abstract

**Importance:**

Limited access to appropriate specialists and testing may be associated with delayed diagnosis and symptom management for patients with early-onset Alzheimer disease and related dementias (ADRDs).

**Objectives:**

To examine rural vs urban differences in diagnostic and symptom management service use among patients with early-onset ADRDs.

**Design, Setting, and Participants:**

This cross-sectional study was conducted using commercial claims from 2012 to 2017. Included patients were those with early-onset ADRDs aged 40 to 64 years, including new patients, defined as those with no claims of ADRDs for 36 months before the first ADRD diagnosis. The likelihood of receiving diagnostic and symptom management services was estimated, with adjustment for individual-level variables associated with health care use. Data were analyzed from February 2021 to March 2022.

**Exposures:**

Rural residence.

**Main Outcomes and Measures:**

Among patients with new, early-onset ADRDs, use of psychological assessment and neuropsychological testing performed at the initial diagnosis (index date) or 90 days or less after the index date and use of brain imaging during the 180 days before the index date were collected. Access to different clinicians on the index date or 90 days or less after the index date was also collected, including visits to primary care physicians and nurse practitioners (PCPs) and specialty visits to psychologists, neurologists, and psychiatrists.

**Results:**

Among 71 799 patients with early-onset ADRD (mean [SD] age, 56.34 [6.05] years; 39 231 women [54.64%]), 8430 individuals had new early-onset ADRDs (mean [SD] age, 55.94 [6.30] years; 16 512 women [56.65%]). There were no statistically significant differences between new patients with early-onset ADRDs in rural vs urban areas in the use of psychological assessments, imaging studies, or visits to neurologists or psychiatrists. However, new patients in rural areas were less likely to receive neuropsychological testing (odds ratio [OR], 0.83; 95% CI, 0.70-0.98) or visit a psychologist (OR, 0.72; 95% CI, 0.60-0.85) compared with patients in urban areas. However, new patients in rural areas with early-onset ADRDs were more likely to have only PCP visits for diagnosis and symptom management compared with those in urban areas (OR, 1.40; 95% CI, 1.19-1.66).

**Conclusions and Relevance:**

This study found that new patients with early-onset ADRDs in rural areas were less likely to receive neuropsychological testing or visit psychologists but more likely to be diagnosed and treated exclusively by PCPs compared with those in urban areas. These findings suggest that efforts, such as clinician education or teleconsultative guidance to PCPs, may be needed to enhance access to specialist services in rural areas.

## Key Points

**Question:**

What are the rural vs urban differences in use of diagnostic and management services at the time of early-onset Alzheimer disease and related dementias (ADRDs)?

**Findings:**

In this cross-sectional study of 71 799 patients with early-onset ADRDs, patients with new early-onset ADRDs in rural areas had fewer neuropsychological tests and fewer visits to clinical psychologists and were more likely to be diagnosed and cared for by primary care physicians and nurse practitioners than those in urban areas.

**Meaning:**

This study found that patients in rural settings with early-onset ADRDs had increased odds of being treated by primary care physicians and nurse practitioners, suggesting that clinician training or consultative guidance to primary care clinicians may be needed to overcome neuroscience workforce deficiencies in rural areas.

## Teaser

This cross-sectional study investigates differences between patients in rural and urban areas in use of cognitive assessment and treatment services for early-onset Alzheimer disease and related dementias.

## Introduction

Alzheimer disease and related dementias (ADRDs) are debilitating conditions that cause loss of memory, cognition, and independent functioning. While most patients with ADRDs are older than age 65 years,^1^ approximately 6% have early-onset ADRDs between ages 30 and 65 years.^2^ Early-onset ADRDs are associated with a more aggressive clinical course, with accelerated cognitive and functional declines compared with later-onset ADRDs.^3,4,5,6^ As a result, patients with early-onset ADRDs may experience rapid cognitive decline and become highly dependent on unprepared family caregivers. Additionally, because of the younger age at inception with early-onset ADRDs, most patients are still in the workforce and continue to have notable family and social responsibilities. Thus, timely diagnosis and management of early-onset ADRDs may help patients preserve daily functioning longer.^1^

Because of atypical symptoms and the young age at beginning stages of disease, diagnosis of early-onset ADRDs can often be challenging. For instance, patients with early-onset ADRDs often present with multidomain cognitive impairments with relative sparing of memory function.^2,7,8,9,10^ This can be associated with unfortunate delays in diagnosis. For example, a study^6^ reported a mean delay of 1.6 years in the diagnosis of early-onset ADRDs compared with late-onset disease. Because of those challenges, early-onset ADRDs may require expertise from relevant specialists, such as neurologists, psychologists, and psychiatrists, for timely diagnosis. These specialists may perform neuropsychological and behavioral assessments that demand special training for administration and interpretation. While primary care physicians and nurse practitioners (PCPs) may play crucial roles in the diagnosis and care of these patients, they often need consultative guidance from specialists regarding diagnostic and symptom management approaches.^11^ Patients with cognitive impairment who are evaluated by specialists also have shorter times to diagnosis and incur lower costs after diagnosis.^12^ Diagnostic assessments can also include advanced imaging studies to rule out medically reversible causes of dementia.^13^ Accurate and early diagnosis of ADRDs can facilitate the development of symptom management plans, allowing caregivers and families to arrange appropriate accommodations for care. Appropriate and timely care plans may help mitigate some of the onerous disease burden from patients and families.^2,6^ If access to specialists with expertise in ADRDs and diagnostic testing is limited, early-onset ADRDs may be misdiagnosed or there may be diagnostic delays.^14,15^

Access to specialty clinicians with requisite expertise in the evaluation of cognitive impairment may be especially challenging in rural areas, where clinician shortages have been well described.^16,17,18^ However, there is a dearth of studies of the use of specialists or the deployment of appropriate tests for early-onset ADRDs in rural areas compared with urban areas. Thus, we used a nationwide claims database from a privately insured population to examine rural-urban differences in use of imaging studies and neuropsychologic assessments at the time of diagnosis of early-onset ADRDs. Our study may provide evidence on rural-urban differences in patterns of care and access to specialist clinicians for the diagnosis and management of early-onset ADRDs. Our findings may be especially relevant for exploring policies to enhance the availability of a specialist workforce and allocate necessary resources for timely diagnosis and symptom management among patients in rural areas with early-onset ADRDs.

## Methods

The Ohio State University Institutional Review Board exempted this cross-sectional study from review and waived informed consent because it used deidentified data. This study followed the Strengthening the Reporting of Observational Studies in Epidemiology (STROBE) reporting guideline.

### Study Design

We conducted a retrospective cross-sectional study using the 2012 to 2018 IBM Watson MarketScan Commercial Claims and Encounters Database.^19^ This nationwide claims database includes information regarding diagnoses, health service procedures, clinician types, and care settings for commercially insured enrollees. The database also contains monthly insurance enrollment information, insurance plan type, age, sex, and rural residency data.

### Identifying Early-Onset ADRD Population

We identified adults aged 40 to 64 years with any ADRD diagnosis in outpatient or inpatient claims in any year during the observation period. To identify ADRD diagnoses, we followed the algorithm that the Centers for Medicare & Medicaid Services (CMS) uses to identify ADRDs in the Medicare population using *International Classification of Diseases, Ninth Revision* (*ICD-9*) and *International Statistical Classification of Diseases and Related Health Problems, Tenth Revision* (*ICD-10*) codes in claims data.^20^ We required individuals to be continuously enrolled for the full calendar year with medical and prescription drug coverage each year.

We identified new patients as those having no claims of ADRDs for 36 months before the first ADRD diagnosis in claims (as the principal diagnosis). CMS uses the 36-month period as the reference window to identify ADRDs in Medicare. To apply this approach, we required new patients to be continuously enrolled during the 3-year look-back period, identifying new patients between 2015 and 2018. We defined the index date of a new patient as the date of the first observed ADRD diagnosis after 3 consecutive years without an ADRD claim. Using data from patients with newly diagnosed early-onset ADRDs, we conducted retrospective cohort analyses of health care use.

### Measures

We measured the prevalence of early-onset ADRDs by determining the number of patients with early-onset ADRDs per 100 000 individuals per year during 2012 to 2018. The denominator included all commercial enrollees, irrespective of diagnosis, in the data who were aged 40 to 64 years and had continuous coverage in a given year between 2012 and 2018. We also measured the incidence of early-onset ADRDs by determining the number of patients newly diagnosed with ADRDs per 100 000 commercially insured individuals in the population at risk of dementia per year during 2015 to 2018.

We followed up new patients for 12 months after the index date and established 4 measures of health care use associated with the principal diagnosis of ADRD among patients with new early-onset ADRDs. With this inclusion criterion, patients who had their first ADRD diagnosis after December 31, 2017, were excluded. We identified use with Current Procedural Terminology (CPT) codes from outpatient claims. We used 2 measures to capture cognitive and functional assessments performed on the index date or 90 days or less after the index date among patients with new early-onset ADRDs: psychological assessment testing, including biopsychosocial assessment and diagnostic psychological tests (CPT codes 90791, 96101, 96102, 96103, 96105, and 96111), and neuropsychological testing (CPT codes 96116, 96118, 96119, 96120, 96132, 96133, 96136, and 96137).

We used another binary measure to examine diagnostic service use of brain imaging during 180 days prior to the index date, including computed tomography scans, magnetic resonance imaging, magnetic resonance angiogram scans, or positron emission tomography scans (CPT codes 70496, 70450, 70460, 70470, 0042T, 70544, 70545, 70546, 70551, 70552, 70553, 70554, 70555, 78608, and 78609). As part of sensitivity analyses, we alternated the observation period of imaging services to 12 months prior to the index date to investigate whether imaging studies were performed within a year prior to diagnosis.

To address access to specialty care, we captured whether a patient with a newly diagnosed early-onset ADRD visited a specialist with expertise in dementia on the index date or 90 days or less after the index date. We created a binary indicator separately for visits to psychologists, neurologists, or psychiatrists. Additionally, we investigated whether a patient with a new early-onset ADRD visited a PCP and none of 3 types of specialists on the index date or 90 days or less after the index date. We used this outcome to evaluate access to specialists and substitution of primary care for specialty care in the diagnosis and management of early-onset ADRDs.

### Statistical Analysis

We summarized characteristics of patients with early-onset ADRDs using descriptive statistics. Then, we modeled the likelihood of receiving diagnostic and treatment services, adjusting for individual-level variables associated with health care use. We estimated a logistic regression model separately for each dependent variable. All *P* values were 2-sided, and results were deemed statistically significant at *P* < .05 at 95% CI. Data were analyzed from February 2021 through March 2022. Data were analyzed using SAS statistical software version 9.4 (SAS Institute).

The key explanatory variable of the analysis was rurality, defined as residing in a nonmetropolitan statistical area (MSA). Thus, rural areas in our study included those outside of an urbanized area with a population of at least 50 000 individuals and its surrounding communities.

Other control variables included age (represented by 5 groups: ages 40-44 years [reference group], 45-49 years, 50-54 years, 55-59 years, and 60-64 years), sex, census region (Northeast, South, Midwest, and West) based on residential state, and enrollee plan characteristics as reflected by plan type, including health maintenance organization (HMO) or point of service (POS), preferred provider organization (PPO) or exclusive provider organization (EPO), high-deductible plan (HDHP), and others. Insurance types were grouped by whether they required PCP referral (HMO or POS), common provider network rules (PPO or EPO), and cost-sharing structures (HDPD).

Lastly, we calculated a risk score for each enrollee based on the Department of Health and Human Services hierarchical condition category (HCC) risk adjustment model developed for commercially insured populations.^21^ This HCC score accounts for age and sex, as well as health conditions associated with *ICD-9* or *ICD-10* diagnoses in a year. It is a proxy for health status, reflecting resource use intensity and potential chronic condition disease burdens. Higher risk scores indicate more severe illness and more complex health care needs, while individuals with a risk score below 1 are considered relatively healthy.

## Results

Among 71 799 patients with early-onset ADRDs at ages 40 to 64 years during 2012 to 2018 (mean [SD] age, 56.34 [6.05] years; 39 231 [54.64%] women), 8430 individuals were new patients (mean [SD] age, 55.94 [6.30] years; 16 512 [56.65%] women). Table 1 describes characteristics of 10 316 patients in rural areas (mean [SD] age, 56.61 [6.23] years; 5633 [54.60%] women) and 61 483 patients in urban areas (mean [SD] age, 55.83 [6.31] years; 34 953 [56.85%] women) with early-onset ADRDs. Prevalence rates of early-onset ADRDs in rural and urban settings were nearly identical. There were 108 patients with early-onset ADRDs per 100 000 privately insured individuals in rural populations, and the prevalence was 107 patients per 100 000 privately insured individuals in urban areas. The incidence rate of patients newly diagnosed with ADRDs was 27 individuals per 100 000 privately insured individuals in rural and urban settings. Mean HCC risk scores were more than 10 for patients in rural and urban areas. Among patients with early-onset ADRDs, 7179 patients (10.00%) had diagnosis of behavioral disturbances in both rural and urban settings.

**Table 1.** Patient Characteristics

Characteristic	Patients, No. (%) (N = 71 799)	
	Rural (n = 10 316)	Urban (n = 61 483)
Early-onset ADRD, No. per 100 000 privately insured individuals		
Prevalence	108	107
Incidence	27	27
Sex		
Women	5633 (54.60)	34 953 (56.85)
Men	4683 (45.40)	26 530 (43.15)
Age, mean (SD), y	56.61 (6.23)	55.83 (6.31)
Age group, y		
40-44	630 (6.11)	4507 (7.33)
45-49	1086 (10.53)	6905 (11.23)
50-54	1461 (14.16)	10 723 (17.44)
55-59	2757 (26.73)	16 600 (27.00)
60-64	4382 (42.48)	22 749 (37.00)
HCC score	1040 (10.08)	6284 (10.22)
Diagnosis of behavioral disturbance	1015 (9.84)	6148 (10.00)
Insurance plan type		
HMO or POS	122 348 (11.86)	12 155 (19.77)
PPO or EPO	641 758 (62.21)	33 109 (53.85)
HDHP or CDHP	1890 (18.32)	11 639 (18.93)
Other	785 (7.61)	4580 (7.45)

Abbreviations: ADRD, Alzheimer disease and related dementia; CDHP, consumer directed health plan; EPO, exclusive provider organization; HCC, hierarchical condition category; HDHP, high-deductible plan; HMO, health maintenance organization; POS, point of service; PPO, preferred provider organization.

**Figure 1.**
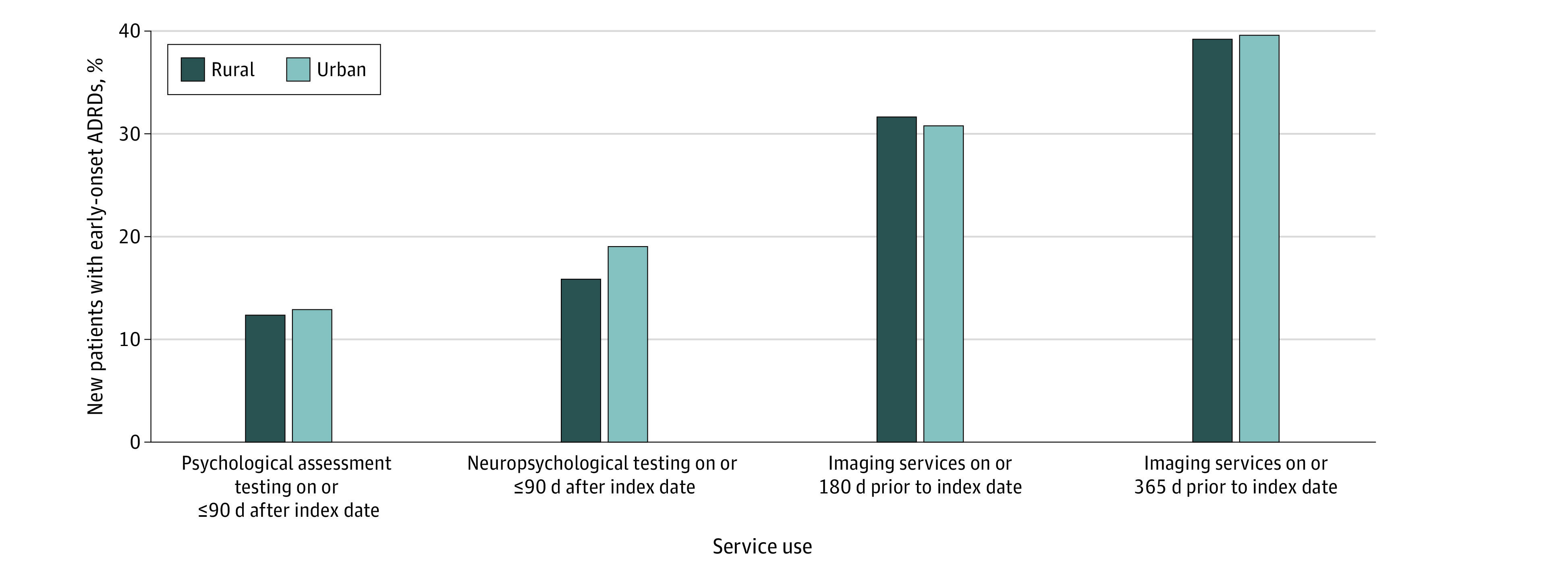
Unadjusted Rural-Urban Differences in Service Use Around Diagnosis Index date indicates date of the first-observed Alzheimer disease and related dementia (ADRD) diagnosis after 3 consecutive years of no ADRD claim. All differences were statistically significant at 95% CI for unadjusted comparison between new patients in rural vs urban areas.

Figure 1 displays the unadjusted rural-urban differences in diagnostic testing and assessments for new early-onset ADRDs. Among 7311 patients in urban areas, there was a higher rate of neuropsychological testing at 90 days or less after the diagnostic index date compared with 1119 patients in rural areas (1385 patients [18.95%] vs 177 patients [15.84%]). Rates of psychological assessment testing or imaging were similar in rural and urban settings.

**Figure 2.**
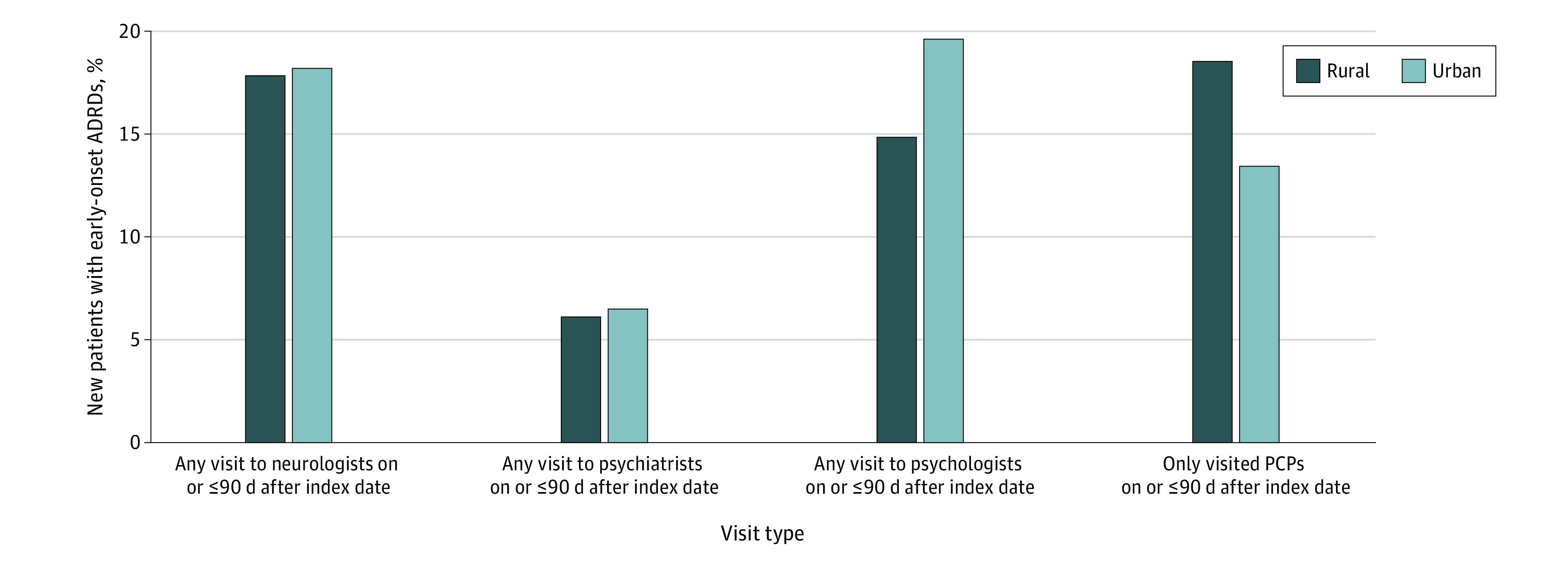
Unadjusted Rural-Urban Differences in Clinician Visits Around Diagnosis Index date indicates the date of first-observed Alzheimer disease and related dementia (ADRD) diagnosis after 3 consecutive years of no ADRD claim. All differences were statistically significant at 95% CI for unadjusted comparison between new patients in rural vs urban areas.

Unadjusted rural-urban differences in clinician visits around diagnosis among patients with newly diagnosed early-onset ADRDs are presented in Figure 2. A similar proportion of patients with early-onset ADRDs in rural and urban areas visited neurologists on the index date or 90 days or less after the index date (198 patients [17.70%] vs 1313 patients [17.96%]). We also found that patterns of visits to psychiatrists were similar in rural and urban areas (67 patients [6.02%] vs 473 patients [6.47%]). However, 163 patients with early-onset ADRDs in rural areas (14.60%) visited a psychologist compared with 1420 patients in urban areas (19.42%). In contrast, more than 18% of patients with early-onset ADRDs in rural areas saw a PCP without visiting other specialists (205 patients [18.32%]), while this was true for 970 patients in urban areas (13.27%).

Table 2 presents results from logit models examining the association of rurality with odds of receiving neurologic assessments and imaging. After controlling for individual patient characteristics, there were no statistically significant differences between patients with early-onset ADRDs in rural vs urban areas in receiving psychological assessments or imaging studies on the index date or 90 days or less afterward. However, patients with early-onset ADRDs in rural areas were significantly less likely to receive neuropsychological testing compared with patients in urban areas (odds ratio [OR], 0.83; 95% CI, 0.70-0.98).

**Table 2.** Logistic Regression Models of Use of Diagnostic Services (N = 8430)

Characteristic	OR (95% CI)^a^			
	Cognitive assessment on or ≤90 d after index date^b^		Imaging services on or before index date^b^	
	Psychological	Neuropsychological	180 d	365 d
Rural residency	0.94 (0.77-1.14)	0.83 (0.70-0.98)	1.01 (0.88-1.15)	0.95 (0.84-1.09)
Male sex	1.02 (0.89-1.16)	0.94 (0.84-1.05)	1.03 (0.94-1.13)	1.00 (0.91-1.09)
Age group, y				
40-44	1 (Reference)	1 (Reference)	1 (Reference)	1 (Reference)
45-49	0.87 (0.66-1.15)	1.05 (0.81-1.37)	1.01 (0.80-1.28)	0.89 (0.72-1.11)
50-54	0.81 (0.63-1.05)	1.17 (0.91-1.41)	1.24 (1.00-1.54)	1.18 (0.97-1.45)
55-59	0.72 (0.56-0.92)	1.12 (0.89-1.41)	1.39 (1.13-1.70)	1.43 (1.18-1.73)
60-64	0.61 (0.48-0.77)	0.97 (0.77-1.22)	1.45 (1.19-1.78)	1.47 (1.22-1.77)
HCC score, per unit increase	0.99 (0.99-1.00)	0.99 (0.98-0.99)	1.01 (1.01-1.01)	1.01 (1.01-1.01)
Census region				
Northeast	1 (Reference)	1 (Reference)	1 (Reference)	1 (Reference)
Northcentral	0.81 (0.66-0.99)	0.92 (0.78-1.09)	1.01 (0.87-1.17)	1.07 (0.93-1.23)
South	1.06 (0.90-1.26)	0.80 (0.69-0.92)	1.07 (0.95-1.22)	1.11 (0.99-1.25)
West	0.52 (0.39-0.67)	0.66 (0.53-0.81)	0.88 (0.74-1.05)	0.95 (0.80-1.12)
Insurance plan type				
HMO	1 (Reference)	1 (Reference)	1 (Reference)	1 (Reference)
PPO or EPO	1.00 (0.84-1.19)	0.92 (0.79-1.06)	1.04 (0.92-1.18)	1.00 (0.89-1.13)
HDHP	0.95 (0.77-1.17)	1.02 (0.86-1.22)	1.11 (0.95-1.29)	1.04 (0.90-1.20)
Other plan	0.94 (0.69-1.27)	0.72 (0.55-0.93)	0.99 (0.80-1.22)	0.88 (0.72-1.07)

Abbreviations: ADRD, Alzheimer disease and related dementia; EPO, exclusive provider organization; HCC, hierarchical condition category; HDHP, high-deductible plan; OR, odds ratio; PPO, preferred provider organization.

^a^ ORs and 95% CIs are presented for all values in logistic models. Regression models are adjusted for individual-level characteristics (age, sex, rurality, insurance plan type, census region, and HCC score).

^b^ Index date is defined as the date of the first-observed ADRD diagnosis after 3 consecutive years of no ADRD claim.

Table 3 contains results of logistic regressions examining access to selected specialist clinicians. We found that patients with newly diagnosed early-onset ADRDs who lived in rural areas were significantly less likely to have visited a psychologist compared with patients who lived in urban areas (OR, 0.72; 95% CI, 0.60-0.85). However, patients in rural areas with new early-onset ADRDs were significantly more likely to have only PCPs involved in their diagnosis and symptom management compared with patients who lived in urban areas (OR, 1.40; 95% CI, 1.19-1.66).

**Table 3.** Logistic Regression Models of Clinician Visits (N = 8430)

Characteristic	OR (95% CI)^a^			
	Visit on or ≤ 90 d after index date^b^			Visited only PCP on or ≤90 d after index date^b^
	Neurologists	Psychiatrists	Psychologists	
Rural residency	0.92 (0.78-1.09)	0.93 (0.72-1.22)	0.72 (0.60-0.85)	1.40 (1.19-1.66)
Male sex	1.15 (1.03-1.29)	0.86 (0.72-1.03)	1.06 (0.95-1.19)	0.99 (0.87-1.12)
Age group, y				
40-45	1 [Reference]	1 [Reference]	1 [Reference]	1 [Reference]
45-49	1.06 (0.75-1.48)	0.78 (0.51-1.21)	0.98 (0.76-1.25)	0.96 (0.69-1.33)
50-54	1.87 (1.39-2.54)	1.10 (0.75-1.61)	0.97 (0.77-1.23)	0.89 (0.65-1.21)
55-59	2.32 (1.74-3.09)	0.98 (0.68-1.42)	0.90 (0.72-1.13)	1.44 (1.09-1.91)
60-64	2.64 (1.99-3.50)	0.96 (0.67-1.38)	0.73 (0.59-0.91)	1.56 (1.18-2.05)
HCC score, per unit increase	0.97 (0.97-0.98)	1.01 (1.01-1.01)	0.99 (0.99-1.00)	1.00 (0.99-1.00)
Census region				
Northeast	1 [Reference]	1 [Reference]	1 [Reference]	1 [Reference]
Northcentral	0.85 (0.71-1.02)	0.96 (0.73-1.27)	0.83 (0.70-0.99)	1.03 (0.84-1.26)
South	1.10 (0.95-1.28)	1.07 (0.85-1.36)	0.80 (0.70-0.92)	1.13 (0.95-1.35)
West	0.86 (0.70-1.06)	0.65 (0.46-0.94)	0.45 (0.36-0.56)	1.27 (1.01-1.59)
Insurance plan type				
HMO or POS	1 [Reference]	1 [Reference]	1 [Reference]	1 [Reference]
PPO or EPO	1.11 (0.95-1.30)	0.72 (0.57-0.91)	0.99 (0.85-1.14)	1.21 (1.01-1.44)
HDHP	1.16 (0.96-1.40)	1.27 (0.99-1.65)	0.88 (0.74-1.06)	1.20 (0.97-1.48)
Other	1.17 (0.91-1.51)	0.70 (0.46-1.05)	0.71 (0.54-0.92)	1.81 (1.39-2.35)

Abbreviations: ADRD, Alzheimer disease and related dementia; EPO, exclusive provider organization; HCC, hierarchical condition category; HDHP, high-deductible plan; HMO, health maintenance organization; OR, odds ratio; PCP, primary care physician; POS, point of service; PPO, preferred provider organization.

^a^ ORs and 95% CIs are presented for all values in logistic models. Regression models are adjusted for individual-level characteristics (age, sex, rurality, insurance plan type, census region, and HCC score).

^b^ Index date is defined as the date of the first-observed ADRD diagnosis after 3 consecutive years of no ADRD claim.

## Discussion

Timely diagnosis and management of early-onset ADRDs can be difficult because patients often present with atypical symptoms and onset at a younger age compared with dementing illnesses in older cohorts. Diagnosis and symptom management among patients with early-onset ADRDs in rural settings can present an even greater challenge. First, in many rural areas, there is a dearth of specialists who have requisite skills to conduct and interpret special diagnostic assessments and supportive treatment for dementing illnesses.^11,15,17,22,23^ Moreover, long travel distances for patients may create access barriers to subspecialty services in rural areas for accurate and timely diagnosis of early-onset ADRDs.^12^ Findings from this cross-sectional study suggest that a more primary care–directed pattern of care occurred among patients in rural areas with newly diagnosed early-onset ADRDs compared with those in urban settings. We found that patients in rural areas were less likely to obtain neuropsychological tests compared with patients in urban areas. We also found that patients in rural areas were less likely to receive care from psychologists. Instead, patients with early-onset ADRDs in rural areas relied more on PCPs for diagnosis and management of ADRDs.

Our study finding that rural residency was associated with lower likelihood of visiting psychologists at 90 days or less after diagnosis warrants special mention. This finding is consistent with the finding that patients in rural areas received fewer neuropsychological tests. These tests are designed to identify possible causes of apparent changes in cognitive function (eg, ADRDs or brain injury). Thus, the tests play a major role in the diagnosis, confirmation, and management of dementia. Neuropsychological tests are usually provided by neuropsychologists with specialized training, which includes preparation to assess levels of cognition, sensory perception, language abilities, abstract reasoning, and other aspects of learning and understanding. Most PCPs are not trained to administer neuropsychological evaluations to characterize behavioral and cognitive functions of patients with early-onset ADRDs. However, a rural practice often lacks access to a clinician with the training of a neuropsychologist. Nearly half of nonmetropolitan areas in the US have no psychologists.^23^ This will likely exacerbate diagnostic delays for early-onset ADRDs in rural areas. In addition, rural residents with early-onset ADRDs may face challenges in having symptoms appropriately managed by clinicians with expertise and experience in cognitive impairment.

While we found that there was limited access to neuropsychological tests among patients with early-onset ADRDs in rural areas, there were no significant differences in use of psychological testing. These tests have notable differences. Neuropsychological tests are more specialized and are used to evaluate different cognitive abilities, including executive function and problem solving. Psychological tests are less specialized and detailed. Our study found fewer visits to psychologists among rural residents but statistically identical levels of psychological testing, suggesting the interesting possibility that other health professionals may have delivered these assessments. While it is highly unlikely that PCPs performed the tests, some rural practices may have clinically licensed social workers who could have been trained in the administration of psychological testing.

Although our study found a reliance on primary care among patients with early-onset ADRDs in rural areas, we did not find significant differences in access to neurologists between rural and urban residents. This was surprising given the more than 4-fold difference between urban and rural areas in the supply of neurologists^24^ There are several potential reasons for this finding. First, our findings suggest that PCPs were referring patients in rural areas with early-onset ADRDs to neurologists or that patients were seeking specialty care on their own. Second, health plans may have maintained more adequate networks of neurologists than psychologists because reimbursements to psychologists have conventionally been low. Moreover, most patients in rural areas may have established the pattern of seeing specialist physicians, such as neurologists, in a medical center even when it required farther travel distances.

For unknown reasons, the prevalence of early-onset ADRDs has increased in recent years.^13^ Given severe clinician shortages in most rural areas, this suggests that community health care leaders and policy makers should explore innovative solutions to deliver needed specialty care to patients with early-onset ADRDs. Increasing diagnosis rates may be associated with increasing costs of care and additional hurdles in the health care system. With a low number of available specialists to treat patients with early-onset ADRDs in rural areas, the most beneficial roles of specialists and PCPs may merit further research. While PCPs provide valuable care for patients in rural areas, these clinicians may need guidance from specialists regarding diagnostic approaches to cognitive impairment.^11^ The recent substantial growth in telehealth during the COVID-19 pandemic represents a prospect to augment the ability to expedite access to clinical psychologists and neuropsychologists in rural areas. Future improvement in broadband internet services may alleviate infrastructure problems, with associated improvements in opportunities to access specialists for residents in rural neighborhoods. Telehealth access to behavioral clinicians may be especially important for communities with strong stigmas against mental health care. Furthermore, building on telehealth care, strategies such as Project Extension for Community Healthcare Outcomes (ECHO) may extend opportunities for PCPs to receive specialist training and consult with a team of specialists from various remote sites via regular teleconferencing.^25^ Integrating behavioral consultation with primary care in rural areas may be associated with decreased disparities in diagnostic care access among patients with early-onset ADRDs in rural areas.

### Limitations

There are several limitations worth noting in this study. First, the CMS algorithm used to identify patients with early-onset ADRDs was developed primarily for the Medicare population. This algorithm includes early-onset and non–early-onset diagnosis codes. We applied all diagnosis codes to our study population because clinicians may not always use early-onset–specific *ICD-9* and *ICD-10* codes. Second, definitions of patients with early-onset or new ADRDs were based on diagnoses in insurance claims data. Our study would miss patients with ADRDs who received medical care paid entirely out of pocket. Similarly, we could not observe income or education levels, factors that may be associated with health care use. Third, we could not identify neuropsychologists, a highly specialized group of psychologists, within all psychologists in our data. Fourth, we could not use a nuanced definition for rurality because the data did not contain geographic information more specific than the level of metropolitan statistical areas.

## Conclusions

This cross-sectional study found that patients with early-onset ADRDs in rural areas had fewer neuropsychological tests and visits to clinical psychologists and were more likely to be diagnosed and treated by PCPs in the 90 days after diagnosis compared with those in urban areas. Given increasing rates of early-onset ADRDs, our findings suggest a need to improve access to neuroscience specialists in rural areas. Clinician training or consultative guidance to PCPs may be viable options to help overcome neuroscience workforce deficiencies in rural areas.
